# Water transport to the core–mantle boundary

**DOI:** 10.1093/nsr/nwab007

**Published:** 2021-01-12

**Authors:** Michael J Walter

## Abstract

Water is transported to Earth's interior in lithospheric slabs at subduction zones. Shallow dehydration fuels hydrous island arc magmatism but some water is transported deeper in cool slab mantle. Further dehydration at ∼700 km may limit deeper transport but hydrated phases in slab crust have considerable capacity for transporting water to the core-mantle boundary. Quantifying how much remains the challenge.

Water can have remarkable effects when exposed to rocks at high pressures and temperatures. It can form new minerals with unique properties and often profoundly affects the physical, transport and rheological properties of nominally anhydrous mantle minerals. It has the ability to drastically reduce the melting point of mantle rocks to produce inviscid and reactive melts, often with extreme chemical flavors, and these melts can alter surrounding mantle with potential long-term geochemical consequences. At the base of the mantle, water can react with core iron to produce a super-oxidized and hydrated phase, FeO_2_H_x_, with the potential to profoundly alter the mantle and even the surface and atmosphere redox state, but only if enough water can reach such depths [1].

Current estimates for bulk mantle water content based on the average H_2_O/Ce ratio of oceanic basalts from melt inclusions and the most un-degassed basalts, coupled with mass balance constraints for Ce, indicate a fraction under one ocean mass [2], a robust estimate as long as the basalts sampled at the surface tap all mantle reservoirs. The mantle likely contains some primordial water but given that the post-accretion Earth was very hot, water has low solubility and readily degasses from magma at low pressures, and its solubility in crystallizing liquidus minerals is also very low, the mantle just after accretion may have been relatively dry. Thus, it is plausible that most or even all of the water in the current mantle is ‘recycled’, added primarily by subduction of hydrated lithospheric plates. If transport of water to the core–mantle boundary is an important geological process with planet-scale implications, then surface water incorporated into subducting slabs and transported to the core–mantle boundary may be a requirement.

Water is added to the basaltic oceanic crust and peridotitic mantle in lithospheric plates (hereafter, slab crust and slab mantle, respectively) at mid-ocean ridges, at transform faults, and in bending faults formed at the outer rise prior to subduction [3]. Estimates vary but about 1 × 10^12^ kg of water is currently subducted each year into the mantle [4], and at this rate roughly 2–3 ocean masses could have been added to the mantle since subduction began. However, much of this water is returned to the surface through hydrous magmatism at convergent margins, which itself is a response to slab dehydration in an initial, and large, release of water. Meta-basalt and meta-sediments comprising the slab crust lose their water very efficiently beneath the volcanic front because most slab crust geotherms cross mineral dehydration or melting reactions at depths of less than 150 km, and even if some water remains stored in minerals like lawsonite in cooler slabs, nearly complete dehydration is expected by ∼300 km [5].

Peridotitic slab mantle may have much greater potential to deliver water deeper into the interior. As shown in Fig. 1a, an initial pulse of dehydration of slab mantle occurs at depths less than ∼200 km in warmer slabs, controlled primarily by breakdown of chlorite and antigorite when slab-therms cross a deep ‘trough’, sometimes referred to as a ‘choke point’, along the dehydration curve (Fig. 1a) [6]. But the slab mantle in cooler subduction zones can skirt beneath the dehydration reactions, and antigorite can transform directly to the hydrated alphabet silicate phases (Phases A, E, superhydrous B, D), delivering perhaps as much as 5 wt% water in locally hydrated regions (e.g. deep faults and fractures in the lithosphere) to transition zone depths [6]. Estimates based on mineral phase relations in the slab crust and the slab mantle coupled with subduction zone thermal models suggest that as much as 30% of subducted water may have been transported past the sub-volcanic dehydration front and into the deeper mantle [4], although this depends on the depth and extent of deep hydration of the slab mantle, which is poorly constrained. Coincidentally, this also amounts to about one ocean mass if water subduction rates have been roughly constant since subduction began, a figure tantalizingly close to the estimated mantle water content based on geochemical arguments [2]. But what is the likely fate of water in the slab mantle in the transition zone and beyond?

**Figure 1.**
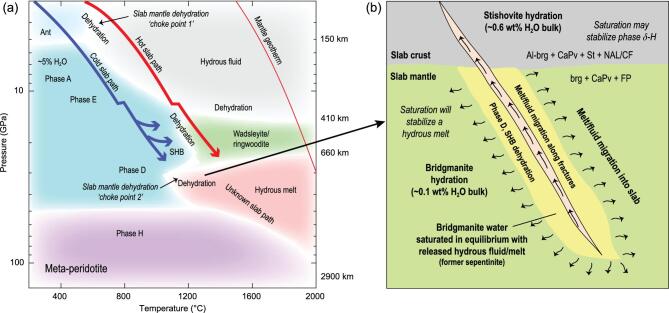
(a) Schematic phase relations in meta-peridotite modified after [6,10,12]. Slab geotherms are after those in [4]. Cold slabs may transport as much as 5 wt% water past ‘choke point 1’ in locally hydrated regions of the slab mantle, whereas slab mantle is dehydrated in warmer slabs. Colder slab mantle that can transport water into the transition zone will undergo dehydration at ‘choke point 2’. How much water can be transported deeper into the mantle and potentially to the core depends on the dynamics of fluid/melt segregation in this region. (b) Schematic showing dehydration in the slab mantle at choke point 2. Migration of fluids within slab mantle will result in water dissolving into bridgmanite and other nominally anhydrous phases with a bulk storage capacity of ∼0.1 wt%, potentially accommodating much or all of the released water. Migration of fluids out of the slab into ambient mantle would also hydrate bridgmanite and other phases and result in net fluid loss from the slab. Conversely, migration of hydrous fluids into the crust could result in extensive hydration of meta-basalt with water accommodated first in nominally anhydrous phases like bridgmanite, Ca-perovskite and NAL phase, but especially in dense SiO_2_ phases (stishovite and CaCl_2_-type) that can host at least 3 wt% water (∼0.6 wt% in bulk crust).

Lithospheric slabs are expected to slow down and deform in the transition zone due to the interplay among the many factors affecting buoyancy and plate rheology, potentially trapping slabs before they descend into the lower mantle [7]. If colder, water-bearing slabs heat up by as little as a few hundred degrees in the transition zone, hydrous phases in the slab mantle will break down to wadsleyite or ringwoodite-bearing assemblages, and a hydrous fluid (Fig. 1a). Wadselyite and ringwoodite can themselves accommodate significant amounts of water and so hydrated portions of the slab mantle would retain ∼1 wt% water. A hydrous ringwoodite inclusion in a sublithospheric diamond with ∼1.5 wt% H_2_O may provide direct evidence for this process [8].

But no matter if slabs heat up or not in the transition zone, as they penetrate into the lower mantle phase D, superhydrous phase B or ringwoodite in the slab mantle will dehydrate at ∼700–800 km due to another deep trough, or second ‘choke point’, transforming into an assemblage of nominally anhydrous minerals dominated by bridgmanite (∼75 wt%) with, relatively, a much lower bulk water storage capacity (< ∼0.1 wt%) [9] (Fig. 1a). Water released from the slab mantle should lead to melting at the top of the lower mantle [10], and indeed, low shear-wave velocity anomalies at ∼700–800 km below North America may be capturing such dehydration melting in real time [11].

The fate of the hydrous fluids/melts released from the slab in the deep transition zone and shallow lower mantle determines how much water slabs can carry deeper into the lower mantle. Presumably water is released from regions of the slab mantle where it was originally deposited, like the fractures and faults that formed in the slab near the surface [3]. If hydrous melts can migrate into surrounding water-undersaturated peridotite within the slab, then water should dissolve into bridgmanite and coexisting nominally anhydrous phases (Ca-perovskite and ferropericlase) until they are saturated (Fig. 1b). And because bridgmanite (water capacity ∼0.1 wt%) dominates the phase assemblage, the slab mantle can potentially accommodate much or all of the released water depending on details of how the hydrous fluids migrate, react and disperse. If released water is simply re-dissolved into the slab mantle in this way then it could be transported deeper into the mantle mainly in bridgmanite, possibly to the core–mantle boundary. Water solubility in bridgmanite throughout the mantle pressure-temperature range is not known, so whether water would partially exsolve as the slab moves deeper stabilizing a melt or another hydrous phase, or remains stable in bridgmanite as a dispersed, minor component, remains to be discovered.

Another possibility is that the hydrous fluids/melts produced at the second choke point in the slab mantle at ∼700 km migrate out of the slab mantle, perhaps along the pre-existing fractures and faults where bridgmanite-rich mantle should already be saturated, and into either oceanic crust or ambient mantle (Fig. 1b). If the hydrous melts move into ambient mantle, water would be consumed by water-undersaturated bridgmanite, leading to net loss of water from the slab to the upper part of the lower mantle, perhaps severely diminishing the slab’s capacity to transport water to the deeper mantle and core. But what if the water released from slab mantle migrates into the subducting, previously dehydrated, slab crust?

Although slab crust is expected to be largely dehydrated in the upper mantle, changes in its mineralogy at higher pressures gives it the potential to host and carry significant quantities of water to the core–mantle boundary. Studies have identified a number of hydrous phases with CaCl_2_-type structures, including *δ*-AlOOH, *ϵ*-FeOOH and MgSiO_2_(OH)_2_ (phase H), that can potentially stabilize in the slab crust in the transition zone or lower mantle. Indeed, these phases likely form extensive solid solutions such that an iron-bearing, alumina-rich, *δ-*H solid solution should stabilize at ∼50 GPa in the slab crust [12], but only after the nominally anhydrous phases in the crust, (aluminous bridgmanite, stishovite, Ca-perovskite and NAL phase) saturate in water. Once formed, the *δ-*H solid solution in the slab crust may remain stable all the way to the core mantle boundary if the slab temperature remains well below the mantle geotherm otherwise a hydrous melt may form instead [12] (Fig. 1a). But phase *δ-*H solid solution and the other potential hydrated oxide phases, intriguing as they are as potential hosts for water, may not be the likely primary host for water in slab crust. Recent studies suggest a new potential host for water—stishovite and post-stishovite dense SiO_2_ phases [13,14].

SiO_2_ minerals make up about a fifth of the slab crust by weight in the transition zone and lower mantle [15] and recent experiments indicate that the dense SiO_2_ phases, stishovite (rutile structure—very similar to CaCl_2_ structure) and CaCl_2_-type SiO_2_, structures that are akin to phase H and other hydrated oxides, can host at least 3 wt% water, which is much more than previously considered. More importantly, these dense SiO_2_ phases apparently remain stable and hydrated even at temperatures as high as the lower mantle geotherm, unlike other hydrous phases [13,14]. And as a major mineral in the slab crust, SiO_2_ phases would have to saturate with water first before other hydrous phases, like *δ-*H solid solution, would stabilize. If the hydrous melts released from the slab mantle in the transition zone or lower mantle migrate into slab crust the water would dissolve into the undersaturated dense SiO_2_ phase (Fig. 1b). Thus, hydrated dense SiO_2_ phases are possibly the best candidate hosts for water transport in slab crust all the way to the core mantle boundary due to their high water storage capacity, high modal abundance and high-pressure-temperature stability.

Once a slab makes it to the core–mantle boundary region, water held in the slab crust or the slab mantle may be released due to the high geothermal gradient. Heating of slabs at the core–mantle boundary, where temperatures may exceed 3000°C, may ultimately dehydrate SiO_2_ phases in the slab crust or bridgmanite (or *δ-*H) in the slab mantle, with released water initiating melting in the mantle and/or reaction with the core to form hydrated iron metal and super oxides, phases that may potentially explain ultra-low seismic velocities in this region [1,10]. How much water can be released in this region from subducted lithosphere remains a question that is hard to quantify and depends on dynamic processes of dehydration and rehydration in the shallower mantle, specifically at the two ‘choke points’ in the slab mantle, processes that are as yet poorly understood. What is clear is that subducting slabs have the capacity to carry surface water all the way to the core in a number of phases, and possibly in a phase that has previously seemed quite unlikely, dense SiO_2_.
